# ASO Author Reflections: Patterns of Metastatic Recurrence of Genetically Confirmed Myxoid Liposarcoma

**DOI:** 10.1245/s10434-023-13380-z

**Published:** 2023-03-27

**Authors:** Pauliina Homsy, Carl Blomqvist

**Affiliations:** 1 Department of Plastic Surgery, University of Helsinki and Helsinki University Hospital, Helsinki, Finland; 2 Department of Oncology, University of Helsinki and Helsinki University Hospital, Helsinki, Finland

Myxoid liposarcomas, constituting around a third of all liposarcomas, differ from other soft tissue sarcomas in their propensity to develop extrapulmonary metastases.^1,2^ The pattern of metastases is, however, not well documented, limiting the ability to propose guidelines for imaging. Moreover, the diagnostic criteria for these tumors have been recently further defined with the identification of the pathognomonic gene translocations: the more common t(12;16)(q13;p11) resulting in fusion of *FUS* and *DDIT3* and the t(12;22)(q13;q12) translocation resulting in *EWSR1:DDIT3* fusion.^3,4^

In the present study, we described the disease course of patients with genetically confirmed myxoid liposarcoma, to our knowledge for the first time.^5^ Interestingly, reviewing our retrospective patient population, we observed that 17% of the patients treated as having myxoid liposarcoma did not harbor a characteristic gene translocation. We also performed a systematic review of literature focusing on the metastatic pattern of myxoid liposarcoma, demonstrating that soft tissues and the abdominal cavity are the most common sites for first metastases, while only around a fifth of the metastases first develop in the lungs.

Our findings have two key implications. Firstly, confirmation of the pathognomonic gene translocation of myxoid liposarcomas is essential. Secondly, chest imaging is inadequate for staging of myxoid liposarcomas, while whole-body imaging can enable the detection of subclinical metastases and thereby improve treatment planning.
